# Epidemiology and diagnosis of metabolic dysfunction-associated fatty liver disease

**DOI:** 10.1007/s12072-024-10704-3

**Published:** 2024-07-05

**Authors:** Yasser Fouad, Mohamed Alboraie, Gamal Shiha

**Affiliations:** 1 Department of Gastroenterology, Hepatology and Endemic Medicine, Faculty of Medicine, Minia University, Minia, Egypt; 2 Department of Internal Medicine, Al-Azhar University, Cairo, Egypt; 3 Egyptian Liver Research Institute and Hospital, Mansoura, Egypt; 4 Hepatology and Gastroenterology Unit, Internal Medicine Department, Faculty of Medicine, Mansoura University, Mansoura, Egypt

**Keywords:** Metabolic dysfunction, Fatty liver, MAFLD, NAFLD, Diagnosis, Epidemiology

## Abstract

The most common chronic liver illness worldwide is metabolic dysfunction linked to fatty liver disease (MAFLD), which is poorly understood by doctors and patients. Many people with this disease develop steatohepatitis, cirrhosis and its consequences, as well as extrahepatic manifestations; these conditions are particularly common if they are linked to diabetes mellitus or obesity. A breakthrough with numerous benefits is the switch from NAFLD to MAFLD in terms of terminology and methodology. The diagnosis of MAFLD is based on affirmative criteria; unlike NAFLD, it is no longer based on exclusion. The diagnosis of MAFLD and the evaluation of steatosis and fibrosis is achieved using liver biopsy and non-invasive laboratory or radiographic techniques. We briefly address the most recent developments in MAFLD epidemiology and diagnosis.

## Epidemiology of metabolic dysfunction-associated fatty liver disease (MAFLD)

The global burden of fatty liver disease has increased noticeably in the past few decades, rising from 21.9% in 1991 to 37.3% in 2019 [1]. Despite being the most common chronic liver disease in the world today, not many people are aware of how common MAFLD is or how important it is to diagnose the condition. In a significant number of people, it can progress to steatohepatitis, cirrhosis and its consequences, particularly if it is accompanied by diabetes mellitus or obesity [2].

An international panel of experts proposed in 2019 to replace the outdated nomenclature of non-fatty liver disease (NAFLD) with metabolic dysfunction-associated fatty liver disease (MAFLD) [3]. Positive criteria (hepatic steatosis with overweight or obesity, type 2 diabetes mellitus, or metabolic dysregulation) are used by the MAFLD diagnostic algorithm as opposed to NAFLD [4]. The regional distribution of MAFLD varies with average worldwide prevalence approximately 33%. Sub-Saharan Africa reported the lowest global prevalence using ultrasound screening of ~ 14%, while South America reported the highest prevalence at 44% [5]. Younger people have a lower prevalence of the disease where it is estimated to be between 8.0 and 16.0% [6].

It should be noted that low degrees of steatosis can lead to incorrect and lower prevalence estimates; this is particularly the case when ultrasound is used as the diagnostic modality [7]. In patients with type 2 diabetes, MAFLD prevalence can reach up to 65%. MAFLD prevalence can also vary depending on the presence of comorbidities [8].

### Natural history of MAFLD

The rs738409 C > G polymorphism in the human patatin-like phospholipase domain-containing-3 (PNPLA3) gene is linked to a genetic propensity to MAFLD. Compared to other genotypes, patients with the GG genotype have a higher chance of acquiring MAFLD. In cirrhosis patients, this polymorphism is also linked to hepatocellular cancer [9, 10].

In 4–7.1% of cases of MAFLD, steatohepatitis may develop, which can progress to cirrhosis within 10 years in a recent large retrospective cohort [5]. Once cirrhosis develops, progression to decompensated cirrhosis and/or liver cancer can ensue [11] (Table 1). Diabetes, increased liver enzymes, and hypertension are some of the risk factors that predict a higher rate of disease progression [12]. Patients with MAFLD have distinctive features associated with hepatocellular carcinoma. HCC in MAFLD has a bad prognosis, a late presentation, and a low response to curative therapy, and it can occur in the absence of cirrhosis.

**Table 1:** Comparison between NAFLD and MAFLD

	NAFLD	MAFLD
Diagnostic approach	Negative based on exclusion	Positive based on inclusion criteria
Criteria of diagnosis	Hepatic steatosis by imaging or biomarkers or histology Plus No excessive alcohol consumption Plus No other causes of hepatic steatosis (e.g., hepatitis C virus, hepatitis B virus, autoimmune liver disease, drugs, Wilson disease, etc.)	Hepatic steatosis detected either by imaging techniques, blood biomarkers/scores, or liver histology, plus (1) Overweight or obese (2) Type 2 diabetes mellitus or (3) Presence of ≥ 2 metabolic risk abnormalities Metabolic risk abnormalities include: (1) Waist circumference ≥ 102/88 cm in Caucasian men and women (or ≥ 90/80 cm in Asian men and women) (2) Blood pressure ≥ 130/85 mmHg or specific drug treatment (3) Plasma triglycerides ≥ 150 mg/dL (≥ 1.70 mmol/L) or specific drug treatment (4) Plasma HDL cholesterol < 40 mg/dL (< 1.0 mmol/L) for men and < 50 mg/dL (< 1.3 mmol/L) for women or specific drug treatment (5) Prediabetes (i.e., fasting glucose levels 100–125 mg/dL (5.6–6.9 mmol/L), or 2-h post-load glucose levels 140–199 mg/dL (7.8–11.0 mmol), or HbA1c 5.7%–6.4%) (6) HOMA-IR ≥ 2.5 (7) Plasma hs-CRP level > 2 mg/L
Other liver disease	No	Concomitant liver disease may be present (Dual aetiology or more)

MAFLD, metabolic dysfunction-associated fatty liver disease; NAFLD, non-alcoholic fatty liver disease; HDL, high-density lipoprotein; HbA1c, glycosylated hemoglobin; HOMA-IR, homeostatic model for assessment of insulin resistance; hs-CRP, high-sensitivity C-reactive protein; BMI, body mass index

## Diagnosis of MAFLD

MAFLD is diagnosed when hepatic steatosis is detected by imaging, non-invasive biomarkers, or liver histology in an individual with evidence of metabolic dysregulation. The criteria for metabolic dysfunction are straightforward and include the presence of Type 2 diabetes mellitus, overweight or obesity, or clinical markers of metabolic dysfunction such as increased waist circumference or abnormal glycemia or lipid profiles. The extent of liver fibrosis can then be assessed by non-invasive methods such as elastography or liver biopsy. To distinguish between MAFLD and dual (or more) aetiology diseases, the patient should be assessed for other liver diseases, particularly viral hepatitis and alcohol use disorder. It should be noted, however, that the presence of another liver disease does not negate the diagnosis of co-existent MAFLD [4]. Table 1 illustrates the distinction between MAFLD and NAFLD (adopted from reference [14]).

## The assessment of fibrosis and steatosis in MAFLD

Steatosis and fibrosis can be evaluated using non-invasive laboratory or radiographic techniques, or liver biopsy with histological evaluation.

### Liver biopsy

Liver biopsy is indicated in cases of MAFLD to confirm a diagnosis in patients with an atypical presentation, in patients within the grey area, to estimate prognosis, and to identify individuals with additional causes for liver disease. A suitable liver specimen can be obtained by performing a percutaneous biopsy using a 16 G or larger needle under ultrasound guidance. An adequate specimen for histological interpretation needs to have ten or more portal tracts and be at least 2 cm in length. The fatty liver inhibition of progression (FLIP) algorithm, the Brunt score, the NAFLD activity score (NAS), and the steatosis, activity, and fibrosis (SAF) score are the commonly used systems to assess MAFLD biopsies. Emerging evidence suggests that the SAF score provides more robust histological assessment [14–16]. Liver biopsy, however, has many limitations such as inter-observer variability, sampling error, cost, and the low but definite risk of complications.

## Interpretation of biopsy findings

Hematoxylin and eosin are used to detect morphological abnormalities, picrosirius red or Mallory's stain is used to detect fibrosis, and Perl's staining is used to diagnose hemosiderosis. The presence of steatosis, portal and lobular inflammation, and hepatocyte ballooning determines the severity of necroinflammation, which is classified as mild, moderate, or severe [17]. The sum of scores for steatosis, ballooning, and lobular inflammation add up to the NAS score. Cases with NAS ≥ 5 are labelled as definite metabolic associated steatohepatitis (MASH), whereas scores of 3 and 4 are borderline. Cases with NAS 0–2 are deemed not-MASH [18]. The inter-observer variability is improved using the SAF (steatosis, activity, and fibrosis) score [19]. For this score, fibrosis is categorised as (0) no fibrosis, (1) perisinusoidal fibrosis, (2) periportal and perisinusoidal fibrosis, (3) bridging fibrosis, and (4) cirrhosis.

### Non-invasive* tests (NITs)*

NITs can be used to diagnose MAFLD, to assess the stage of disease, and to monitor treatment response. The diagnosis of MAFLD depends on identifying hepatic steatosis either by histology or imaging. While abdominal ultrasonography is often adequate for detecting hepatic steatosis, its sensitivity is low for steatosis < 20% [21]. The controlled attenuation parameter (CAP) can be obtained simultaneously with liver stiffness measurement (LSM) by vibration controlled transient elastography (VCTE). This method of detecting steatosis, however, is more qualitative than quantitative; if the CAP score is higher than a threshold, steatosis is considered to be present; nevertheless, the extent of steatosis does not correlate with higher readings. Although 248 dB/M is the most widely used cut-off, several studies have indicated higher optimal cut-offs, such as 288 dB/M and 302 dB/M. The choice of probe has an impact on CAP values and the M versus XL probes may yield different ideal cut points for the diagnosis of fatty liver. Theoretically, CAP can track alterations in hepatic steatosis over time [21–23].

When it comes to measuring liver fat, magnetic resonance imaging (MRI) methods are considered the gold standard. Very small amounts of liver fat can be detected by magnetic resonance spectroscopy (MRS) and MRI-proton density fat fraction (MRI-PDFF). Currently, clinical research studies are the main indications for MRS and MRI-PDFF, given the cost and the need for specialised equipment [24].

Several simple scores have been suggested as substitutes for evaluating hepatic steatosis in large population studies. The fatty liver index (FLI) incorporating BMI, waist circumference, triglycerides, and GGT is one of the most frequently used scores. Its utility was recently confirmed in a large group of patients with MAFLD [26, 27]. A score known as the ultrasonographic fatty liver indicator (US FLI) can be used to rule out steatohepatitis when ultrasonographic features are closely inspected [27]. However, despite the recent notable progress in ultrasonography-based methods, it remains difficult to identify the inflammatory aspects of steatohepatitis using this technology. Further research is required to precisely identify the utility of the most advanced ultrasonographic techniques while reducing costs and increasing feasibility [28].

The degree of liver fibrosis in MAFLD has the strongest relationship with morbidity, liver-related outcomes, and death. NITs for fibrosis can be divided into three categories: specific fibrosis biomarkers, imaging biomarkers, and simple fibrosis scores [28, 29].

Simple fibrosis scores: these scores are inexpensive, reproducible, and widely validated. They involve clinical and routine laboratory parameters including FIB-4 (fibrosis-4 index), APRI (aspartate aminotransferase (AST)-to-platelet ratio index, and the NFS (NAFLD fibrosis score). Patients can be defined as being at low or high risk for advanced fibrosis for each score according to the following cut-offs: FIB-4 (1.30 and 2.67), APRI (0.5 and 1.5), and NFS (< − 1.455 and > 0.67611). These scores are well suited for use as an initial assessment in primary-care or resource-poor settings [31–34]. The inclusion of liver enzymes in the models represents a significant constraint as their levels might be normal in the presence of fibrosis. Further, liver enzymes vary with age [34].

In specialised settings, fibrosis markers can direct patient care. Among these is the enhanced liver fibrosis (ELF) panel, which demonstrated good overall accuracy in several observational studies and clinical trials. The N-terminal type III collagen peptide (Pro-C3) is another biomarker that indicates production of type III collagen. The ADAPT algorithm has an area under the receiver-operating characteristics curve of 0.87 for advanced fibrosis and incorporates age, T2DM, Pro-C3, and platelet count. In low-risk population-based, and tertiary hospital cohorts, it has good diagnosis accuracy for advanced fibrosis [35–37].

A novel nomograph-based non-invasive model was found to be more accurate in diagnosing patients with MAFLD and determining their risk of significant fibrosis than APRI, NFS, and FIB-4. This model combined the waist-to-height ratio (WHTR), hyaluronidase (HA), serum collagen type III N-telopeptide (P3NP), chitinase 3-like protein 1 (CHI3L1), and CK-18 M65 [38]. The following five variables were then used to create an MLA model in 2021: serum procollagen type III (PC-III), albumin–globulin (A/G) ratio, BMI, collagen type IV (IV–C), and AST. MLA has the highest diagnosis accuracy in comparison to other diagnostic models [39] in patient cohorts [35–37].

Liver stiffness measurement (LSM) by VCTE is a commonly used method that is favoured over biopsy. The majority of patients with MAFLD can have their stiffness measured using the XL probe. For good quality, there must be a minimum of 10 measurements, more than 60% of which must be valid, and the ratio of the median valid LSM to IQR must not be greater than 0.3. Compared to VCTE, magnetic resonance elastography is more accurate, but its wider application is constrained by cost and availability [40–43]. Shear wave elastography is another option for measuring liver stiffness and has diagnostic performance similar to VCTE for advanced hepatic fibrosis. The FAST score combines AST with CAP and liver stiffness measurement by VCTE and achieves a c-statistic of 0.74–0.95 for the detection of steatohepatitis with fibrosis [44].

## Diagnosis of MAFLD cirrhosis

Because hepatic steatosis can disappear with progression to cirrhosis, patients with cirrhosis who meet the other diagnostic criteria for MAFLD are considered to have MAFLD associated cirrhosis [4]. In individuals with MAFLD, fibrosis severity can be reliably assessed by LSM, which can also be utilised to identify cirrhosis. Even though cirrhosis in some instances can be identified using ultrasonography, liver fat can mask the diagnosis. Thus, it is imperative to assess MAFLD cirrhosis using methods other than ultrasonography [45] LSM < 10 kPa rules out compensated advanced chronic liver disease, while LSM > 15 kPa is strongly suggestive and 10–15 kPa is suggestive [46]. Patients with MAFLD who have thrombocytopenia and/or LSM > 20–25 kPa are likely to have clinically significant portal hypertension and should have variceal screening by endoscopy. On the other hand, MAFLD patients with LSM > 15 kPa ought to be under HCC surveillance. The prognosis of MAFLD patients may also be evaluated by LSM, and the risk of death increases with increasing LSM [46, 47].

In most instances, liver biopsy is not indicated and will not alter patient management in the context of MAFLD cirrhosis. As per the 2022 APASL guidelines [48], individuals with cirrhosis who do not have conventional histology and who fit the following descriptions can be diagnosed with cirrhosis related to MAFLD: evidence of metabolic risk factors, either past or present, that satisfy the requirements for diagnosing MAFLD with at least one of the following: (1) Documentation of MAFLD on a previous liver biopsy. (2) Historical documentation of steatosis by hepatic imaging. A history of past alcohol intake should be taken into consideration as patients may have dual disease etiology with alcohol use disorder.

### Diagnosis and impact of MAFLD in the setting of other liver diseases

MAFLD has the potential to coexist with other liver disease, including primary hemochromatosis, alcohol-related liver disease (ARLD), chronic hepatitis B virus infection (CHB), and chronic hepatitis C virus infection (CHC). A diagnosis of mixed or dual etiology liver disease should be made if the patient meets the criteria for a diagnosis of MAFLD plus one or more other, less common causes of fatty liver, either at baseline or during follow-up. Examples of these include long-term use of steatogenic medications, HCV genotype 3 infection, and Wilson disease [48].

Notably, MAFLD may synergistically cause liver cirrhosis or possibly the development of HCC in individuals with ARLD and CHB [49, 50]. As a result, patients with MAFLD should have any concurrent liver diseases thoroughly assessed and managed as appropriate. Moreover, underlying systemic metabolic dysfunction in MAFLD may increase the risk of cardiometabolic events in patients with other liver disease.

More research is needed to understand the natural history, therapeutic responsiveness, and prognosis of MAFLD patients with ARLD. The diagnosis of dual etiology fatty liver disease will be aided by a thorough history obtained during the patient interview, including information about past and present alcohol consumption. Recent research has produced a wealth of information challenging the so-called “safe limits” for alcohol use in the context of MAFLD [51, 52], since even modest alcohol consumption may raise the risk of cirrhosis and cancer [53–55] and reduce the rate at which steatohepatitis improves. Thus, it is wise to counsel MAFLD patients to abstain from alcohol or to consume it sparingly.

In studies from some parts of the world, it has been estimated that 38% of people with CHC have concomitant MAFLD [56]. In individuals with CHC, MAFLD considerably increases the rate at which liver disease progresses, the responsiveness to treatment, and the emergence of certain extrahepatic complications. It has also been shown that eliminating hepatitis C virus with direct-acting antiviral medications, or earlier with interferon therapy, reduces adverse liver outcomes and insulin resistance. A recent study from Thailand revealed that MAFLD was independently linked to an elevated risk of advanced liver fibrosis in CHB patients, suggesting that MAFLD may hasten the progression of this liver disease. Another study showed that in individuals with CHB, MAFLD alone enhanced the probability of developing HCC by 7.3-fold [57, 58]. Poor outcomes and chronically abnormal liver tests in persons with CHB and/or CHC infection following virological suppression or sustained virological response are pointers to the likely existence of MAFLD. Treatment for MAFLD in this group should, therefore, be prioritised [59–62].

In conclusion, metabolic dysfunction-associated fatty liver disease is the most prevalent liver disease in the world today. The identification of this disease is aided by the recently developed diagnostic framework, which will expand knowledge of the condition's natural history and management.
